# Navigating Mixed-Neurotype Dialogue: Environmental and Affective Influences on Autistic Speakers

**DOI:** 10.1177/13623613261469909

**Published:** 2026-08-03

**Authors:** Zachary A. Miller, Caitlin M. Conner, Judy C. Chang, Carla A. Mazefsky

**Affiliations:** 1 University of Pittsburgh, PA, USA; 2 Michigan State University, East Lansing, USA; 3 University of Pittsburgh School of Medicine, PA, USA

**Keywords:** autism, double empathy theory, mixed-neurotype communication, emotions, online survey

## Abstract

In this reflective report study, we examined communication challenges for autistic adults by examining the influences of emotional state and environmental context across neurotype conversation partners. Using the Neurotype Communication and Assistive Technology Survey (NCATS), we surveyed 165 autistic adults (18–35 years old) and analyzed 154 responses to capture both quantitative ratings and qualitative descriptions of past contextual conversations. The majority of the participants noted that environmental factors, such as crowdedness, noise levels, and familiarity, at least somewhat impacted their communication. Autistic adults reported that noisy or unpredictable settings were associated with word loss, scripting, and social withdrawal, while quiet and familiar spaces facilitated openness and authentic self-expression. In addition, participants stated that conversations with allistic (non-autistic) partners required more conscious effort, social masking, and cognitive load than conversations with autistic partners, consistent with the Double Empathy Problem. Finally, survey responses revealed that negative emotions such as stress, frustration, and anxiety may worsen communication difficulties regardless of the conversation partner’s neurotype, whereas positive emotions such as happiness and calmness were found to improve fluency, confidence, and intersubjectivity. These results indicate that partner neurotype, emotional states, and other environmental factors can affect communication challenges for autistic adults.

**Lay Abstract:**

In this study, autistic adults reflected on their conversational experiences and described what helped communication go well and what made it more difficult. We asked 165 autistic adults (18–35 years old) to complete a survey about how they communicate in different situations based on people and places and received 154 responses. We focused on how communication changed whether the other person is autistic or allistic (non-autistic). Within that, we focused on two main factors: the type of environment they were in (e.g., home, work, school) and how their emotions (feeling happy, sad, angry) affected their communication. The majority of autistic adults said that the place they were in at least somewhat affected their communication. Autistic adults said that loud or unpredictable places often made communication harder, leading to things like losing words, repeating scripts, or pulling back from the conversation. Quiet and familiar spaces, on the contrary, made it easier to share thoughts openly. Talking with allistic people usually took more effort and sometimes led to masking, or hiding one’s natural way of communicating. This fits with the Double Empathy Problem, which says that communication struggles often come from mismatched understandings between autistic and allistic people. Emotions were also important. Stress, anxiety, or frustration made conversations harder, while happiness and calmness made conversing easier and improved confidence. Overall, our findings suggest that reducing sensory stress, supporting emotional well-being, and encouraging mutual understanding can make conversations better for both autistic and allistic people.

## Introduction

Communication differences and difficulties have been consistently conceptualized as a core characteristic of autism (Banks et al., 2024; Cummins et al., 2020; Kimura et al., 2020; Koegel & Ashbaugh, 2017; Strömberg et al., 2021). Autistic individuals exhibit a broad range of speech and language abilities, from limited or no spoken language, to the use of alternative and augmentative communication, to highly articulate expression. Yet, autistic people often encounter challenges in pragmatic language use, social reciprocity, and conversation maintenance (Ying Sng et al., 2018).

Communication challenges in autism are often described as unilateral deficits, placing most of the burden on autistic individuals to adapt to the norms of allistic (non-autistic) social expectations. Milton (2012; Milton et al., 2022) proposed the Double Empathy Problem (DEP), which posited that communication challenges are not solely a characteristic of autistic individuals but are instead interactional, arising from the contrast between autistic communication styles and the prevailing allistic-based communication world (Milton, 2012; Wadge et al., 2019). From this perspective, both parties may struggle to accurately interpret and respond to each other’s social cues, thereby complicating attempts at mutual understanding.

Previous studies have shown that allistic observers detect atypical patterns in autistic individuals’ interactions. Conversely, allistic speakers may not recognize how their own communicative style contributes to cross-neurotype misunderstandings. (Heasman & Gillespie, 2018, 2019; Jones et al., 2023; Rifai et al., 2021). This concept is often described as mismatched-neurotype communication, wherein autistic and allistic individuals experience disproportionate difficulties relative to neurotype-matched pairs (Geelhand et al., 2024). Within mismatched-neurotype communication, some studies have suggested that groups composed solely of autistic participants may communicate more efficiently with one another than in mixed-neurotype groups (Crompton et al., 2020); however, subsequent replication work has not consistently reproduced this effect (Crompton et al., 2025). While environmental and contextual factors, such as social setting, are known to heighten communication mismatches, a comprehensive understanding of their impact is still lacking (Oates et al., 2024). In addition, researchers have observed that autistic adults tend to alter their communicative behaviors, even unconsciously, when interacting with allistic partners, possibly in an attempt to adhere to perceived social expectations (Cook et al., 2022; Geelhand et al., 2024). However, such adaptations do not always improve understanding and can add stress, emotional, and mental health burdens for autistic individuals (Crompton et al., 2020a; Field et al., 2024; Mandy, 2019; McQuaid et al., 2024).

Moreover, studies indicate that autistic individuals report higher levels of social anxiety and greater difficulty in interactions with allistic individuals relative to interactions with other autistic people (Cage & Troxell-Whitman, 2019; Pickard et al., 2020). These challenges are especially salient during early adulthood, a developmental period marked by increasing social demands, including transitions to higher education, employment, and independent living, where expectations for complex and sustained social communication intensify. During this time, autistic individuals may be required to navigate a broader range of social environments and interpersonal dynamics, often leading to greater reliance on compensatory strategies such as social masking and increased cognitive effort during interactions with allistic individuals (Podesta & Bosman, 2025). These difficulties may lead to increased frustration and negative emotions like sadness or anger when communication breaks down or expectations are unmet during interactions with allistic individuals (Black et al., 2023; Strömberg et al., 2022). Conversely, there are limited findings of positive emotions’ impact on communication, demonstrating an indicated need for positive emotion contexts. Overall, emotional state is thought to influence communication by increasing cognitive load and disrupting social processing, suggesting it is a critical factor to consider alongside neurotype differences (Gaigg, 2012).

The current study aimed to assess the perceived impacts of conversational partner neurotype, setting, and emotional state on autistic adults’ communication. The first aim sought to understand communication challenges across experiences with allistic and autistic conversational partners, as well as across settings. In accordance with prior research, we hypothesized that the participants would report greater communication challenges in conversations with allistic individuals compared to conversations with autistic individuals, regardless of the conversation’s environment. The second aim delved into the emotional state associations of cross-neurotype communication for autistic individuals, assessing how autistic adult’s emotional states impacted their communication styles in mismatched and matched-neurotype interactions. We hypothesized that autistic individuals would experience more communication challenges during negative emotional states, while their communication skills would improve during positive emotional states. We also hypothesized that cross-neurotype communication would present greater communication challenges compared to matched-neurotype communication in all displayed emotional states.

## Methods

### Participants and Recruitment

Participants included autistic adults (18–35 years old, inclusive) with a self identification^1^ of autism as well as a reading level of sixth grade or higher to ensure that they would be able to fully comprehend all the questions. Advertisements and flyers for the survey were distributed among autism and neurodiversity advocacy organizations, including posters being placed around universities as well as public speaking engagements with undergraduate classes and club organizations. The survey was also listed on a national autism study list from the autism Science Foundation to reach a wider audience across the United States. To ensure no fraudulent behavior, a ReCAPTCHA score was added to each response with a required score of 0.7 or higher. Participants were offered compensation of up to $100 for completing the survey. This compensation was randomized, ranging from $5 to $15, with one participant selected for the full $100. Following a 3-month recruitment period, the survey gathered 165 responses. Eleven responses (6.7%) were disqualified due to less than 50% of the survey being completed. The remaining 154 responses were included in both quantitative and qualitative analyses.

### Measures and Procedures

We designed the Neurotype Communication and Assistive Technology Survey (NCATS) to address identified gaps in the literature concerning matched- and mismatched-neurotype communication for autistic individuals. Demographic questions concerning gender, race, ethnicity, socioeconomic status, educational background, age, residency, and service use were also collected. The survey questions were iteratively refined based on feedback from four autistic adults. The final version of the NCATS consisted of 53 questions, including 43 multiple-choice (10 demographic questions and 33 communication-based questions) and 10 short-answer questions (see Supplemental Table 1).

Communication-based items were organized into four domains: environment, interpersonal familiarity, partner neurotype, and emotional state. Each domain followed the same two-step structure. First, participants rated a one-sentence statement on a 5-point Likert-type scale (1 = “not at all/none” to 5 = “all the time/a great deal”); for example, “My surroundings affect my ability to communicate.” Second, an open-ended follow-up invited participants to describe how their communication shifted (e.g., loss of words, masking, greater fluency), ensuring qualitative depth alongside the quantitative rating. For the neurotype domain, the Likert-type items were repeated across specific relationship contexts, such as family, friends, strangers, and authority figures, so that matched- and cross-neurotype effects could be compared directly. The emotional state block of questions further disaggregated affect by valence: participants indicated whether negative emotions improved, worsened, or left unchanged their communication and answered the same for positive emotions. They then rated how strongly emotion influenced communication when interacting with allistic versus autistic partners, providing a direct matched-/cross-neurotype comparison for emotional effects. This mirrored structure across domains clarifies that every major topic was assessed for both matched- and mixed-neurotype interactions, allowing coherent cross-domain analyses of factors that facilitate or hinder autistic adults’ communication.

The survey was approved by an institutional review board with an exempt status. The NCAT survey was deployed online via the Qualtrics Survey Software (Qualtrics, 2005) and was estimated to take a maximum of 60 min.

### Data Analysis

No preregistration was completed for the analyses conducted in this study. Multiple-choice survey data were analyzed using descriptive statistics. The normality of each dataset was assessed with Kolmogorov–Smirnov tests. Wilcoxon signed-rank tests were then employed to determine if significant differences existed between matched versus mismatched-neurotype interactions in the varying survey item contexts. Spearman rank correlation coefficients were calculated to explore associations between survey items related to the communication effects of environments and emotions.

Statistical analyses were performed using GraphPad Prism version 10.0.0 for macOS (GraphPad, 2023). In addition, chi-square tests were conducted in StataSE 18 (StataCorp, 2023) to investigate the influence of demographic factors (gender, socioeconomic status, and race) on communication effects.

In addition, we conducted a content analysis of the responses to the 10 short-answer survey questions. Each participant’s typed responses to the open-ended questions were combined into an individual text file utilizing MATLAB (MathWorks, 2023) developed script and uploaded into NVivo 14 (Lumivero, 2025) for coding. All participants’ responses to each question were coded using an open, editing approach in an iterative fashion (Crabtree & Miller, 2023). Codes were then examined for patterns, categories, and themes regarding communication and emotion changes. We used an integrative mixed-methods approach to convert our codes related to communication experiences into quantitative variables that we then used to perform descriptive statistics (Hochwald et al., 2023; Zickmund et al., 2015).

## Results

### Demographics

The NCAT survey participants took on average 12.57 min (*SD* = 6.71; outliers removed using the 1.5×IQR criterion) to complete at least 50% of the survey, while completion times across the 154 responses (with no outliers removed) ranged from approximately 3.58 to 1,364.72 min. As shown in Table 1, the participant pool was predominantly white (66.3%) and female-identifying (49.1%). In addition to demographic characteristics, participants reported using a range of communication modalities. Verbal and text-based methods were most commonly endorsed, including text messaging (*n* = 121, 76.6%), telephone (*n* = 111, 70.3%), messaging platforms (*n* = 104, 65.8%), and email (*n* = 99, 62.7%). In contrast, support-based and augmentative and alternative communication -related (AAC) strategies were used by a smaller proportion of participants (combined: *n* = 50, 31.6%).

**Table 1.** Participant Demographics.

Variable	*N*	%
Race		
White	106	66.3
Black/African American	35	21.9
American Indian or Alaska Native	6	3.8
Asian	10	6.3
Native Hawaiian or Pacific Islander	1	0.6
Hispanic/Latinx	2	1.3
Sex assigned at birth		
Male	53	34.4
Female	96	62.3
Intersex	0	0
Prefer not to say/No response	5	3.2
Gender identity		
Male	57	35
Female	80	49.1
Non-binary/third gender	16	9.8
Agender/no gender	5	3.1
Unsure/questioning	3	1.8
Transgender/genderqueer/other	2	1.2
Annual income level		
Less than $49,999	111	72.1
$50,000–$99,999	12	7.8
More than $100,000	2	1.3
Prefer not to say	29	18.8

### Environmental Impacts on Communication

#### Quantitative Findings

Overall, 56.4% of participants reported that environmental factors at least somewhat impacted their communication abilities. Spearman correlation analyses demonstrated that greater perceived environmental impact was associated with increased communication adaptation. A moderate positive correlation was observed between environmental impact and communication changes with allistic friends (ρ = .485, *p* < .001), and a weaker positive correlation with allistic individuals more broadly (ρ = .365, *p* < .001). A Williams test for dependent correlations indicated that the association with allistic friends was significantly stronger than with allistic individuals overall (*t*(146) = 1.98, *p* = .025, one-tailed). A strong positive correlation was also observed between adjustments made when interacting with allistic friends and adjustments made in allistic interactions overall (ρ = .65, *p* < .001).

Further, environmental communication difficulty (e.g., difficulty finding words or loss of speech) was associated with increased use of text-based communication methods, including email (ρ = .31, *p* = .0015), text messaging (ρ = .30, *p* = .0028), and AAC-related strategies (*ρ* = .26, *p* = .0215). Other modalities showed weaker, non-significant trends, while telephone communication showed no meaningful association (ρ = −.02, *p* = .7838).

#### Qualitative Findings

A total of 88 participants (57.1%) provided qualitative responses describing how their communication varied across environments. As summarized in Table 2, communication patterns differed systematically by setting. Busy and public environments were most frequently associated with quietness, loss of words, and verbal communication difficulties. Familiar and personal environments were associated with more natural, expressive, and open communication. Unfamiliar environments showed increased communication difficulty and loss of words, while work and school settings were associated with anxiety, communication difficulty, and increased scripting and social masking.

**Table 2.** Frequency (%, *n*) of Mentioned Communication Differences in Different Environments – Qualitative Themes Describing Participants’ Reported Communication Experiences in Busy/Public, Familiar/Personal, Unfamiliar, and Work/School Settings.

Content segment	Busy/public setting	Familiar/personal setting	Unfamiliar setting	Work/school setting
Anxiety, Fear, and Overwhelm	2 (6.1%)	0 (0.0%)	1 (7.7%)	2 (18.2%)
Verbal Communication Difficulties	14 (42.4%)	2 (12.5%)	7 (53.8%)	2 (18.2%)
Natural-Expressive-Open	1 (3.0%)	7 (43.8%)	0 (0.0%)	3 (27.3%)
Quiet/Loss of Words	17 (51.5%)	2 (12.5%)	6 (46.2%)	2 (18.2%)
Scripting and Social Masking	1 (3.0%)	0 (0.0%)	1 (7.7%)	3 (27.3%)
Talkative or Repetitive	4 (12.1%)	3 (18.8%)	0 (0.0%)	1 (9.1%)

*Note.* Percentages represent the proportion of participants who mentioned each theme within a given setting, calculated relative to the total number of responses for that setting.

These patterns were reflected in participant responses. One participant noted: “The more overwhelming the stimuli around me, the harder it is for me to concentrate on my words and properly convey what I’m trying to say . . .” (Participant 22). Another described work-related challenges: “I find it more difficult to communicate at work because I have to try harder and worry more about how I come across” (Participant 10).

### Neurotype and Relationship Contexts

#### Quantitative Findings

Participants reported modifying their communication based on both the neurotype of their communication partner and the relationship context. At least moderate communication changes (⩾3 on a 5-point Likert-type scale) were reported by 30.5% of participants in general interactions, 41.6% in interactions with friends, and 37.3% in interactions with family members. These findings were based on 91 responses (59.1%). In family interactions specifically, just under half of the 109 participants who responded reported no communication adjustments between autistic and allistic relatives, while a slight majority reported at least some change.

#### Qualitative Findings

A total of 131 participants (85.1%) provided qualitative responses describing how their communication differed across interaction partners. As shown in Table 3, communication with autistic individuals was commonly described as direct, specific, and efficient, with themes of mutual intersubjectivity and authentic self-expression. In contrast, interactions with allistic individuals were more frequently associated with misunderstandings, particularly related to vague wording, figurative language, tone, and social cues. Close relationships (friends and family) were associated with more expressive and open communication, whereas interactions with unfamiliar individuals were characterized by shyness, loss of words, and non-speaking moments. Participants highlighted these contrasts in detail. One participant stated: “Communicating with my autistic family members is . . . more direct . . . and avoids ambiguity . . .” (Participant 117). Another described challenges with allistic communication styles: “I struggle with . . . passive aggressive ways of communicating . . . I have to do mental gymnastics . . .” (Participant 129).

**Table 3.** Common Qualitative Themes based on Communication Partner – Most Frequently Reported Qualitative Themes in Interactions with Autistic Friends, Allistic Friends, Close Friends/Family, and Strangers/Unfamiliar People.

Interaction context	Most frequent themes	*n*	%
Autistic friends	Mutual intersubjectivity	35	24.5
	Authentic self-expression	30	21.0
	Spontaneous, casual, direct speech	27	18.9
Allistic friends	Communication misunderstandings (vague wording, figurative language)	36	20.2
	Social-expression misunderstandings (sarcasm, facial cues, tone)	35	19.7
Close friends/family	Expressive, open communication	36	18.8
Strangers/unfamiliar people	Shyness, loss of words, non-speaking moments	30	17.0

### Authority Contexts

Of the 137 participants (89.0%) who responded to this item, 62.3% (*n* = 96) reported difficulty communicating with individuals in positions of authority. These challenges were most commonly attributed to communication difficulties (*n* = 35), feelings of nervousness or anxiety (*n* = 18), and experiences of being misunderstood or judged. One participant described this as:

“I am nervous and feel like I have to put on even more of a show with authority figures, which severely limits how well I can communicate, as my thought processes are taken up with how I am performing rather than the conversation at hand.” (Participant 10)

### Emotions’ Impact on Communication

#### Quantitative Findings

As shown in Table 4, 53.3% of participants (*n* = 82) reported that their communication was frequently influenced by their emotional state (“Often” or “Very much”), while 34.4% (*n* = 53) reported little to no influence, and 12.3% (*n* = 19) were unsure or did not respond. Negative emotional states were most commonly associated with worsened communication (*n* = 81, 52.6%), compared to improvements (*n* = 7, 4.5%) or no change (*n* = 16, 10.4%), with 32.5% (*n* = 50) unsure or not responding. In contrast, positive emotional states were associated with improved communication in 33.1% of participants (*n* = 51), no change in 20.8% (*n* = 32), and worsened communication in 14.9% (*n* = 23), with 31.2% (*n* = 48) unsure or not responding.

**Table 4.** Impact of Emotional State on Communication – Frequency of Participants Reporting Changes in Communication Ability under Positive and Negative Emotional States.

Survey prompt	*n*	%
Does your ability to communicate change based on your emotional state?		
Frequently influenced (“Often” + “Very much”)	82	53.3
Occasionally/no influence (“Not at all,” “A little,” “Neutral”)	53	34.4
Unsure / no answer	19	12.3
When experiencing a negative emotion, your communication . . .		
Improves	7	4.5
Stays the same	16	10.4
Worsens	81	52.6
Unsure/no answer	50	32.5
When experiencing a positive emotion, your communication . . .		
Improves	51	33.1
Stays the same	32	20.8
Worsens	23	14.9
Unsure / no answer	48	31.2

**Figure 1.**
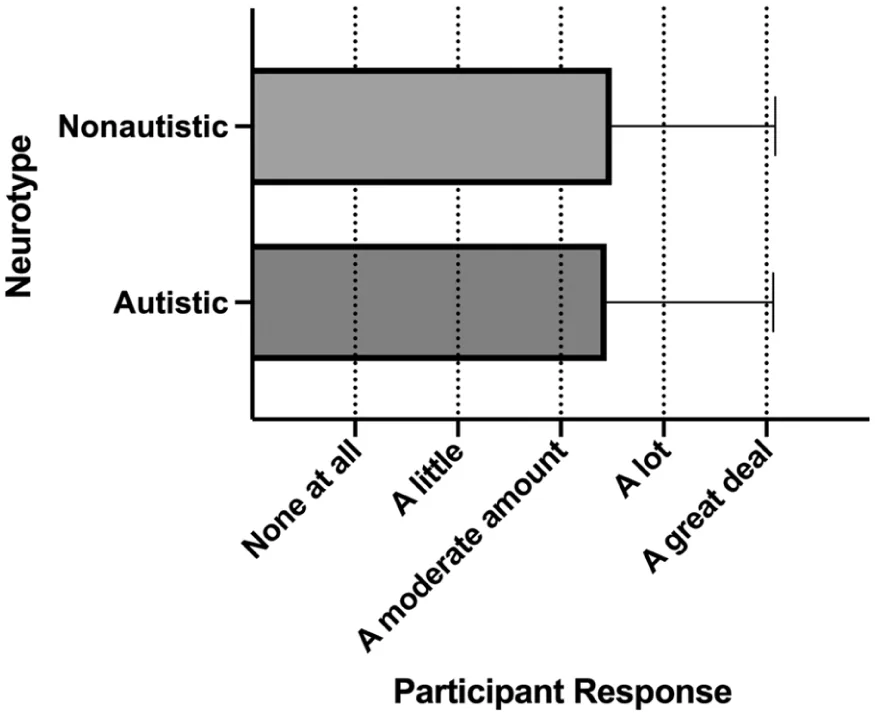
Emotions’ impact on communication based on partner’s neurotype – Frequency of participants reporting changes in communication ability under positive and negative emotional states.

A Wilcoxon signed-rank test indicated no significant difference in the impact of emotions on communication between autistic and allistic interaction contexts (*n* = 154,*W* = 185.0, *p* = .8558; Figure 1). A Spearman correlation demonstrated that participants who experienced greater emotional impact in autistic interactions were also more likely to report greater impact in allistic interactions (ρ = .4694, *p* < .0001).

To examine whether emotional variability was associated with communication modality, additional Spearman correlation analyses were conducted. No communication modalities remained statistically significant after Bonferroni correction, although sign language showed a nominal positive association (ρ = .19, *p* = .024). Other modalities demonstrated weak and non-significant relationships, suggesting that emotional influences on communication are not strongly associated with specific communication modalities.

#### Qualitative Findings

A total of 114 participants (74.0%) provided qualitative responses describing how emotional state influenced communication. As detailed in Table 5, negative emotional states were most frequently associated with loss of words, withdrawal, and reduced verbal communication. Specifically, participants experiencing upset, overwhelmed, or negative emotional states reported loss of words or withdrawal in 94.0% of coded instances (*n* = 63), with additional reports of social masking (*n* = 13, 19.4%) and sensory overload (*n* = 11, 16.4%). Similarly, anxious, afraid, or embarrassed states were associated with loss of words or withdrawal in 78.3% of instances (*n* = 18) and social masking in 21.7% (*n* = 5).

**Table 5.** Participant Contexts of Emotions’ Effects on Communication – Qualitative Categorization (*n*, %) of How Specific Emotions (e.g., Anxiety, Frustration, Calm, Happiness) Influenced Communication Style (Difficulty, Withdrawal, Masking, Expressiveness).

Emotion	Loss of words/quiet/withdrawn	Social mask	Sensory overload	Expressive/talkative	No change
Afraid/Anxious/Embarrassed	18, 78.3%	5, 21.7%	1, 4.3%	3, 13.0%	0, 0.0%
Angry/Frustrated	7, 53.8%	1, 7.7%	1, 7.7%	1, 7.7%	0, 0.0%
Calm/Relaxed/Secure	0, 0.0%	0, 0.0%	0, 0.0%	8, 100.0%	0, 0.0%
Happy/Excited/Positive	10, 15.2%	1, 1.5%	2, 3.0%	59, 89.4%	3, 4.5%
Intense Emotions	15, 78.9%	0, 0.0%	3, 15.8%	4, 21.1%	0, 0.0%
Upset/Overwhelmed/Negative	63, 94.0%	13, 19.4%	11, 16.4%	3, 4.5%	0, 0.0%

In contrast, positive emotional states were associated with increased expressiveness and communication fluency. Participants reporting happy or excited emotional states demonstrated expressive or talkative communication in 89.4% of instances (*n* = 59), with comparatively fewer reports of withdrawal (*n* = 10, 15.2%). Calm or relaxed states were uniformly associated with expressive communication (*n* = 8, 100.0%). Intense emotional states, regardless of valence, were also associated with communication disruption, including withdrawal (*n* = 15, 78.9%) and sensory overload (*n* = 3, 15.8%). These patterns are reflected in participant responses. For example, one participant noted:

When I’m having a bad emotion, sometimes my brain feels like it moves too slowly or like the rest of the world is too far away (as though I’m in a tunnel) to get the right words out in the right order. (Participant 107)

In contrast, another participant stated: “When I am in more positive moods, I tend to talk more, speak louder, speak more openly.” (Participant 15)

## Discussion

This cross-sectional survey study looked to deepen our understanding of the role of environment and emotions in communication dynamics for autistic adults across mixed-neurotype interactions. The results suggest that a communication partner’s neurotype may shape autistic adults’ perceived communication experiences. Participants qualitatively reported greater communication difficulties in mismatched-neurotype conversations and decreased intersubjectivity compared to matched-neurotype conversations. This pattern aligns with existing literature, which reports increased social masking and conformity in cross-neurotype interactions (Cook et al., 2022; Miller et al., 2021). Autistic individuals often report expending considerable cognitive effort adapting their communication style to meet allistic expectations, a process that can be emotionally and psychologically taxing (Ai et al., 2022; Angulo et al., 2019). Participants often described having to “beat around the bush” and engaging in superficial communication, further reflecting perceived communication challenges in cross-neurotype interactions. These findings align with the DEP by suggesting perceived communication difficulties in autistic-allistic communication.

In addition, participants reported experiencing communication difficulties when interacting with authority figures like supervisors and teachers, as revealed in the survey. This aligns with broader literature on employment and education challenges faced by autistic adults, where navigating social hierarchies and implicit workplace expectations often impedes professional success (Syriopoulou-Delli et al., 2024; Szechy et al., 2024). Participants reported that they felt misunderstood or judged for their direct communication style, struggling to understand communication differences between supervisors and acquaintances, which participants described as contributing to perceived communication breakdowns. Despite increased awareness of neurodiversity, many workplaces lack sufficient accommodations and understanding for autistic employees (Waisman-Nitzan et al., 2021; Wen et al., 2023).

In contrast, participants reported experiencing communication benefits across relationships with friends, family, and strangers. These findings are supported by literature demonstrating that matched-neurotype communication is more facilitated and less effort-intensive (Williams et al., 2021). Previous literature has found that autistic individuals communicate more effectively with other autistic individuals and reported that autistic adults feel more understood and accepted in same-neurotype interactions (Crompton et al., 2020b; Heasman & Gillespie, 2019). Participants in our study echoed this, reporting feeling more “seen” and “heard” in matched-neurotype conversations, which participants interpreted as mutual understanding and improved communication.

Comfort and familiarity with the conversation partner were reported to influence how participants perceived their communication ability. Participants reported the highest communication ease with autistic family members and the lowest with allistic friends and strangers. This suggests that both neurotype alignment and familiarity may be important factors in shaping perceived communication experiences. Other prior literature supports that familiarity reduces the cognitive load of social interactions by enabling reliance on established patterns (Morrison et al., 2020), and when paired with a matched neurotype, it improves communication facilitation substantially (Heasman & Gillespie, 2019). The combination of these factors, neurotype and familiarity, may jointly contribute to reduced perceived communication breakdown and enable autistic individuals to express themselves authentically.

Communication modality also emerged as an important contextual factor associated with differences in reported communication experiences. Participants who endorsed non-speaking or supported modalities, including text-based communication and AAC-related strategies, reported greater effects of their surroundings on communication ability, whereas speaking-based modalities such as telephone showed little to no association with environmental communication effects. This suggests that individuals who rely on non-speaking modalities may report greater vulnerability to environment-related communication disruptions, such as difficulty finding words, loss of speech, fogginess, or reduced fluency. These findings align with prior work suggesting that autistic communication preferences and abilities vary by modality, with many individuals reporting that real-time spoken communication can be especially demanding (Howard & Sedgewick, 2021). Importantly, these results do not imply that non-speaking modalities cause greater communication difficulty; rather, they suggest that people whose communication is more environmentally variable may be more likely to rely on alternative, text-based, or AAC-supported methods to maintain communication access.

In addition, varying sensory (e.g., loud, busy, quiet) and environmental contexts (e.g., home, work, school) were reported to affect communication ability, though our participants indicated that these effects are less pronounced than those of neurotype or familiarity. However, our participants also reported difficulty discerning what is contributing to their communication differences. Some previous work has been done to confirm that the environment does influence autistic individual’s communication skills (Louis-Delsoin et al., 2024; O’Connor et al., 2024), but more work is needed in this area.

### Emotional States’ Effects on Communication

Results from this study suggest a major role of emotions in shaping perceived communication ability in shaping the perceived communication abilities of autistic individuals. Our findings suggest that increased positive emotions were associated with reports of more intersubjective communication, while the presence of negative emotions was associated with greater perceived communication difficulties, which is consistent with research on emotion dysregulation’s effect on communication in autism (Samson et al., 2014).

This pattern also aligns with the Yerkes-Dodson Law, which posits that an optimal level of arousal improves performance, while excessive arousal impairs it (Hanoch & Vitouch, 2004; Keith et al., 2019; Yerkes & Dodson, 1908). A moderate level of positive emotional stimulus seems to facilitate communication among participants, as evidenced by increased expressiveness and talkativeness during positive states. However, excessive emotional intensity, positive or negative, may be associated with sensory overload and emotional dysregulation, contributing to perceived communication difficulties. Current research shows that emotion dysregulation and sensory overload in autism disrupt communication by overloading sensory processing and impairing executive functioning, causing difficulties in articulating thoughts or maintaining conversational flow (Keluskar et al., 2021).

Contrary to our hypothesis, the study found no significant difference in the impact of emotions on communication based on the neurotype of the communication partner. Although it was hypothesized that negative emotions would disproportionately impair mismatched-neurotype communication due to DEP, this was not supported. This finding suggests that participants may perceive the neurotype of the communication partner as less relevant. One possible explanation is that strong negative emotions may lead to cognitive overload. Research indicates that cognitive overload in autism, stemming from emotional intensity or sensory demands, can result in conversational difficulties, such as becoming temporarily non-speaking or non-verbal, as cognitive resources are depleted (Green et al., 2015, 2018).

### Limitations

The interpretations in this research are shaped by several potential limitations. A significant challenge was participants’ accuracy in recalling emotions and their subsequent impact on social communication. Many participants commented on struggling to identify their emotions in specific conversations retroactively, which therefore led to many unclear short-answer responses. This difficulty was likely compounded for those who self-identified with or otherwise presented with alexithymia, a condition characterized by difficulty identifying and describing emotions, which may have skewed self-report of emotions. Alexithymia is also known to be more prevalent among autistic individuals, which may further amplify challenges in accurately recalling and reporting emotional experiences (Kinnaird et al., 2019). Identifying situational factors influencing communication during intense emotional states was also challenging. Numerous variables, such as environmental noise, social context, or the partner’s behavior, could contribute to communication dysregulation, making it hard to isolate emotions as the sole cause. Retrospective self-reporting may also lack precision in disentangling these influences.

This study also did not account for the emotional expressions of the communication partner, which may also affect autistic adults’ communication abilities. Autistic individuals have varying degrees of emotional understanding, often showing heightened emotional empathy (Shalev et al., 2022), but potentially struggling to recognize others’ emotional cues. These differences in emotion recognition can lead to greater emotional dysregulation, where the emotional tone of a conversation partner amplifies the autistic individual’s own emotional response, further impacting communication (Brett et al., 2024). For example, if an allistic partner displays frustration, an autistic individual might inherently mirror this emotion, exacerbating communication difficulties in the dyad. Despite these limitations, this aspect of the survey suggests an interplay between emotional states and social communication in autistic individuals.

In addition, self-report bias may arise when participants attempt to align their responses with what they perceive researchers expect, potentially underestimating or overestimating the frequency of communication difficulties. In addition, despite using ReCAPTCHA, there is a risk that some survey participants were computer bots or allistic rather than genuine autistic participants. Such infiltration could skew data by inflating completion rates or misrepresenting real experiences (Bottini et al., 2025; Griffin et al., 2022). In addition, a key limitation of this study is the limited characterization of the participants. Because the study was designed to preserve participant privacy and the Institutional Review Board (IRB)-approved protocol did not include collection or verification of detailed clinical information, we did not collect detailed diagnostic or trait-level data (e.g., autistic traits, co-occurring conditions such as attention-deficit/hyperactivity disorder (ADHD), dyslexia, social anxiety, or alexithymia), nor did we systematically assess the effects of self-identification versus formal diagnosis or gender. As a result, we are unable to determine how these factors may have influenced communication experiences or contributed to variability in the findings. Selection bias is also possible because recruitment strategies targeted advocacy organizations, universities, and online platforms, thus not capturing the experiences of autistic adults who have less access to these networks (Rubenstein & Furnier, 2021; Russell et al., 2019). Demand characteristics can influence how participants respond if they infer the study’s goals and adjust their answers accordingly. Regarding external validity, the narrow age range of autistic adults (18–35) and a predominantly white, female-assigned sample limit the generalizability to broader populations, including older adults, adolescents, or those from other cultural backgrounds. Finally, diagnostic variability, where the study included both self-identified and clinically diagnosed autistic individuals, adds another dimension of heterogeneity that may shape how participants perceive and report communication challenges (Lombardo et al., 2019).

### Future Directions

Future research building on this study’s findings could employ longitudinal designs to track how communication strategies evolve over time, especially as autistic adults transition across life stages. More detailed qualitative approaches, such as ethnographic observations or in-depth interviews, could capture nuanced data on conversational patterns and real-world strategies used to mitigate breakdowns in various settings (O’Reilly et al., 2016). In addition, moment-to-moment changes in mood and speech can be clarified using ecological momentary assessments, partner ratings, and wearable physiology. This data can help understand how spikes in physiological arousal or bursts of reported positive and negative affect trigger or ease breakdowns in mixed-neurotype dialogue. Building on these findings, experimental work can test whether teaching co-regulation techniques, adjusting sensory stressors, or intentionally boosting positive emotion via intervention improves communicative reciprocity for autistic and allistic partners. Intervention studies that teach explicit cross-neurotype communication strategies to allistic individuals might investigate whether structured guidance in directness, clarity, and emotional support reduces misunderstandings.

## Conclusion

This study investigated the ways in which environmental contexts, emotional states, and the neurotype of the conversation partner may converge to shape interactions for autistic adults. In line with DEP, findings suggest that communication breakdowns are influenced by a reciprocal lack of alignment between autistic and allistic speakers. Findings showed that communication adaptability was most pronounced in cross-neurotype interactions, particularly with allistic friends and strangers, whereas autistic-autistic exchanges were characterized by openness and mutual understanding. Environmental context further shaped communication quality, as participants in familiar, calm spaces reported greater fluency and comfort than those in loud or unpredictable settings. In addition, while positive emotions appear to promote clarity and openness, negative affect can undermine verbal fluency in both matched and mismatched-neurotype exchanges. Together, these results highlight that communication for autistic adults is not simply a function of language ability but a dynamic interaction between sensory context, emotional regulation, and relational familiarity. By recognizing these multidimensional influences, future interventions and communication frameworks can more effectively promote mutual understanding and reduce the cognitive and emotional demands placed on autistic communicators.

## Supplemental Material

sj-docx-1-aut-10.1177_13623613261469909 – Supplemental material for Navigating Mixed-Neurotype Dialogue: Environmental and Affective Influences on Autistic Speakers Supplemental material, sj-docx-1-aut-10.1177_13623613261469909 for Navigating Mixed-Neurotype Dialogue: Environmental and Affective Influences on Autistic Speakers by Zachary A. Miller, Caitlin M. Conner, Judy C. Chang and Carla A. Mazefsky in Autism

## Notes

1. Self-identification of being autistic with no given clinical diagnosis or assessment for autism.
