# Supplementary material for: Navigating Mixed-Neurotype Dialogue: Environmental and Affective Influences on Autistic Speakers
Source: Autism. 2026 Aug 3;30(9):2415–26. doi: 10.1177/13623613261469909 (PMC13494137; doi:10.1177/13623613261469909)
Supplement: Supplementary file 1 [file sj-docx-1-aut-10.1177_13623613261469909.docx]

Supplemental Table 1. Survey Short Answer Questions. The listed questions were asked to the participants in descending order. Question numbers with a letter following indicate that it was a nested question.

| Question Number | Question Description |
| --- | --- |
| *21A* | Please describe how your communication varies (regarding your environment). |
| *22A* | Please describe how your communication varies (regarding your relationship with the conversation partner). |
| *23B* | Please describe how your communication is different with your autistic family member(s). |
| *24* | Do you face any challenges in mutual understanding between you and your nonautistic family member(s)? Please explain. |
| *25A* | Please describe how your communication varies with your nonautistic friends versus your autistic friends. |
| *26* | Do you encounter any challenges with you and your nonautistic friends understanding each other? Please explain. |
| *27A* | Please describe how your communication varies between autistic and nonautistic people. |
| *28A* | Please describe how your communication varies with the person in power. |
| *30* | Please describe in your own words how your emotional state can impact your communication ability. (For example, if you are upset versus happy versus afraid, do you communicate the same or differently in these instances). |
| *35A* | Please describe how your assistive technology can be improved (i.e. reduced cost, easier interface, more communication features, emotion detection, conversation facilitation, conversation suggestions, etc.). |

Supplemental Table 2. Quantitative Participant Responses to Communication-based Questions. The descriptive statistics (n and %) are shown for each communication-based question asked in the NCATS.

| *Variable* | *n* | *%* |
| --- | --- | --- |
| **In general, how would you describe your ability to understand speech under good conditions (when you're having a great day)?** |  |  |
| I usually can understand speech well. | 131 | 85.1% |
| I often have a hard time understanding what people say. | 20 | 13.0% |
| I understand very little spoken language. | 2 | 1.3% |
| Did not answer | 1 | 0.6% |
|  |  |  |
| **In general, how would you describe your ability to understand speech in your regular daily life?** |  |  |
| I usually can understand speech well. | 112 | 72.7% |
| I often have a hard time understanding what people say. | 39 | 25.3% |
| I understand very little spoken language. | 1 | 0.6% |
| Did not answer | 2 | 1.3% |
|  |  |  |
| **In general, how would you describe your ability to speak under good conditions (when you're having a great day)?** |  |  |
| I usually can speak well. | 104 | 67.5% |
| I can speak but often have a hard time saying what I want to say, or I say things I don't mean to say. | 47 | 30.5% |
| I have a very hard time speaking (for example, I only can use short phrases). | 3 | 1.9% |
| Did not answer | 0 | 0.0% |
|  |  |  |
| **In general, how would you describe your ability to speak in your regular daily life?** |  |  |
| I usually can speak well. | 90 | 58.4% |
| I can speak but often have a hard time saying what I want to say, or I say things I don't mean to say. | 59 | 38.3% |
| I have a very hard time speaking (for example, I only can use short phrases). | 5 | 3.2% |
| Did not answer | 0 | 0.0% |
|  |  |  |
| **How would you describe your ability to read under good conditions (when you're having a great day)?** |  |  |
| I cannot read | 2 | 1.3% |
| I can read some words and short sentences | 13 | 8.4% |
| I can read at a basic level | 35 | 22.7% |
| I can read at an advanced level | 104 | 67.5% |
| Did not answer | 0 | 0.0% |
|  |  |  |
| **How would you describe your ability to write or type under good conditions (when you're having a great day)?** |  |  |
| I cannot write or type | 1 | 0.6% |
| I can write or type a few words | 9 | 5.8% |
| I can write or type a few short sentences | 40 | 26.0% |
| I can write or type at an advanced level | 104 | 67.5% |
| Did not answer | 0 | 0.0% |
|  |  |  |
| **How often is speech a reliable form of communication from you?** |  |  |
| Rarely or none of the time | 1 | 0.6% |
| A little of the time | 5 | 3.2% |
| About half the time | 33 | 21.4% |
| Most of the time | 62 | 40.3% |
| All or almost all the time | 53 | 34.4% |
| Did not answer | 0 | 0.0% |
|  |  |  |
| **When you need to use something other than speech to communicate, what do you do?** |  |  |
| I type or write | 107 | 49.3% |
| I point on a letterboard | 5 | 2.3% |
| I use picture icons | 17 | 7.8% |
| I use sign language | 22 | 10.1% |
| A supporter helps me get my thoughts out | 7 | 3.2% |
| A supporter interprets my signs, gestures, or behaviors | 8 | 3.7% |
| A supporter communicates for me | 8 | 3.7% |
| I am not able to communicate using something other than speech | 38 | 17.5% |
| Other | 5 | 2.3% |
|  |  |  |
| **Which of these things can you reliably use to communicate?** |  |  |
| Telephone | 107 | 18.9% |
| Video-conference | 71 | 12.5% |
| Text messaging | 117 | 20.6% |
| Email | 95 | 16.8% |
| Messing platforms | 100 | 17.6% |
| Social media platforms | 76 | 13.4% |
| Other | 1 | 0.2% |
|  |  |  |
| **Do your surroundings or environment (i.e. home, school, work, etc.) affect your ability to communicate (e.g., repeating phrases, difficulty finding the right words, loss of words, fogginess, more fluent, etc.)?** |  |  |
| Not at all | 48 | 31.2% |
| A little bit | 19 | 12.3% |
| Sometimes | 45 | 29.2% |
| A lot | 33 | 21.4% |
| All the time | 9 | 5.8% |
| Did not answer | 0 | 0.0% |
|  |  |  |
| **How much does your communication ability vary based on how well you know someone?** |  |  |
| Not at all | 44 | 28.6% |
| A little | 23 | 14.9% |
| A moderate amount | 36 | 23.4% |
| Often | 28 | 18.2% |
| Very often | 23 | 14.9% |
| Did not answer | 0 | 0.0% |
|  |  |  |
| **Are any of your immediate family members autistic? (Biological/adoptive parent(s), biological/full/step/half siblings, biological/adoptive children, etc.)** |  |  |
| Yes | 51 | 33.1% |
| Maybe | 46 | 29.9% |
| No | 28 | 18.2% |
| Unsure | 29 | 18.8% |
| Did not answer | 0 | 0.0% |
|  |  |  |
| **Is communicating different with your autistic family member(s) versus your nonautistic family members?** |  |  |
| Yes | 19 | 37.3% |
| Sometimes | 9 | 17.6% |
| No | 23 | 45.1% |
|  |  |  |
| **Do you change how you communicate when talking with your nonautistic friends versus autistic friends?** |  |  |
| Not at all | 65 | 42.2% |
| A little | 21 | 13.6% |
| A moderate amount | 28 | 18.2% |
| Often | 20 | 13.0% |
| Very often | 16 | 10.4% |
| Did not answer | 4 | 2.6% |
|  |  |  |
| **Does your ability to communicate change based on if you speak with someone autistic versus nonautistic?** |  |  |
| Not at all | 68 | 44.2% |
| A little | 34 | 22.1% |
| A moderate amount | 24 | 15.6% |
| A lot | 13 | 8.4% |
| A great deal | 10 | 6.5% |
| Did not answer | 5 | 3.2% |
|  |  |  |
| **Are there any challenges you and the person in power have in understanding each other?** |  |  |
| Not at all | 58 | 37.7% |
| A little | 26 | 16.9% |
| A moderate amount | 34 | 22.1% |
| A lot | 19 | 12.3% |
| A great deal | 12 | 7.8% |
| Did not answer | 5 | 3.2% |

Supplemental Table 3. Quantitative Participant Responses to Emotion-based Questions. The descriptive statistics (n and %) are shown for each emotion-based question asked in the NCATS.

| *Variable* | *n* | *%* |
| --- | --- | --- |
| **Does your ability to communicate change based on your emotional state? (such as how happy, sad, upset, or excited you feel at any given moment).** |  |  |
| Not at all | 23 | 16.2% |
| A little | 15 | 10.6% |
| Neutral/Stays the same | 14 | 9.9% |
| Often | 27 | 19.0% |
| Very much | 44 | 31.0% |
| Unsure | 14 | 9.9% |
| Did not answer | 5 | 3.5% |
|  |  |  |
| **Does your communication improve or worsen if you are experiencing a negative emotion (such as frustration, overwhelm, stress, anger, or sadness, etc)?** |  |  |
| Improve | 7 | 4.9% |
| Stay the same | 15 | 10.6% |
| Worsen | 70 | 49.3% |
| Unsure | 22 | 15.5% |
| Did not answer | 28 | 19.7% |
|  |  |  |
| **Does your communication improve or worsen if you are experiencing a positive emotion (such as happiness, excitement, feeling proud, etc)?** |  |  |
| Improve | 46 | 32.4% |
| Stay the same | 28 | 19.7% |
| Worsen | 20 | 14.1% |
| Unsure | 20 | 14.1% |
| Did not answer | 28 | 19.7% |
|  |  |  |
| **If you are interacting with a non-autistic person, how much do your emotions impact your communication ability?** |  |  |
| Not at all | 28 | 19.7% |
| A little | 12 | 8.5% |
| Neutral/Stays the same | 24 | 16.9% |
| Often | 31 | 21.8% |
| Very much | 26 | 18.3% |
| Unsure | 15 | 10.6% |
| Did not answer | 6 | 4.2% |
|  |  |  |
| **If you are interacting with another autistic person, how much do your emotions impact your communication ability?** |  |  |
| Not at all | 20 | 14.1% |
| A little | 21 | 14.8% |
| Neutral/Stays the same | 25 | 17.6% |
| Often | 30 | 21.1% |
| Very much | 23 | 16.2% |
| Unsure | 18 | 12.7% |
| Did not answer | 5 | 3.5% |
|  |  |  |
| **Do your emotions impact your communication more or less if the other person is not autistic as compared to autistic?** |  |  |
| More | 33 | 23.2% |
| Less | 20 | 14.1% |
| No difference | 48 | 33.8% |
| Unsure | 35 | 24.6% |
| Did not answer | 6 | 4.2% |

Supplemental Table 4. Correlation Strengths of Environmental Influence on Communication in Varying Contexts - Spearman correlation coefficients (ρ) showing associations between perceived environmental impact and communication changes with autistic and allistic partners.

| **Variable Comparison** | **Correlation Coefficient* (ρ)** |
| --- | --- |
| Environmental impact vs. communication changes with allistic friends | 0.49 |
| Environmental impact vs. communication changes with allistic individuals overall | 0.37 |
| Adjustments with allistic friends vs. adjustments in allistic interactions generally | 0.65 |

*All correlation coefficients (ρ) were significant (p < 0.001).

Supplemental Table 5. Common Qualitative Themes based on Communication Partner - Most frequently reported qualitative themes in interactions with autistic friends, allistic friends, close friends/family, and strangers/unfamiliar people.

| **Interaction Context** | **Most Frequent Themes (n)** |
| --- | --- |
| Autistic friends | - Mutual intersubjectivity (35) - Authentic self-expression (30) - Spontaneous, casual, direct speech (27) |
| Allistic friends | - Communication misunderstandings (vague wording, figurative language) (36) - Social-expression misunderstandings (sarcasm, facial cues, tone) (35) |
| Close friends/family | - Expressive, open communication (34) |
| Strangers/unfamiliar people | - Shyness, loss of words, nonspeaking moments (30) |
